# Carbon-Stimulated Bioaugmentation Enhances Thermogenesis, Lignocellulose Degradation, and Humification in Low-Temperature Cattle Manure Composting

**DOI:** 10.3390/microorganisms14051077

**Published:** 2026-05-10

**Authors:** Mengke He, Doudou Jin, Yaowei Chi, Xianzhong Ma, Shunping Zhang, Ruiren Zhou, Shaohua Chu, Pei Zhou, Dan Zhang

**Affiliations:** 1 School of Agriculture and Biology, Shanghai Jiaotong University, Shanghai 200240, China; mengkh@163.com (M.H.);; 2 Key Laboratory of Urban Agriculture, Ministry of Agriculture and Rural Affairs, Shanghai 200240, China; 3 Shanghai Yangtze River Delta Eco-Environmental Change and Management Observation and Research Station, Ministry of Science and Technology, Ministry of Education, Shanghai 200240, China; 4 Shanghai Urban Forest Ecosystem Research Station, National Forestry and Grassland Administration, Shanghai 200240, China; 5 Chongming Agricultural Environment Field Science Observation and Research Station, Ministry of Agriculture and Rural Affairs, Shanghai 200240, China; 6 Inner Mongolia Research Institute, Shanghai Jiaotong University, Hohhot 010052, China

**Keywords:** low temperature composting, cattle manure, initiator, lignocellulose, microbial community

## Abstract

Low ambient temperatures severely restrict the start-up efficiency and microbial bioconversion of livestock manure during aerobic composting. To overcome this “cold-start” barrier, this study investigated the coupled effects of an easily accessible carbon source (molasses) and functional microbial inoculants (*Streptomyces griseorubens* JSD-1 and *Paenarthrobacter nitroguajacolicus* LDT1-8) on cattle manure composting. Results demonstrated that the combined strategy significantly expedited thermogenesis, achieving a peak temperature of 62.1 °C and extending the thermophilic phase (>50 °C) by 2 days. This enhanced microbial activity accelerated organic matter stabilization, increasing cellulose and hemicellulose degradation by 44.0% and 49.3%, respectively, and boosting humic acid content by 33.4% in treatment T7 (molasses + JSD-1 + LDT1-8). Amplicon sequencing revealed that the amendments reshaped microbial community structure, selectively enriching lignocellulose degradation and humification-driving taxa (e.g., Actinobacteriota and Mycothermus), leading to a more robust and modular metabolic network. Redundancy analysis confirmed that this directed succession was primarily driven by organic matter degradation and humic fraction accumulation. Overall, the combined application of molasses and microbial inoculants promoted temperature rise, lignocellulose degradation, and humification by reshaping microbial community structure, providing an effective strategy for improving cattle manure composting efficiency under low-temperature conditions.

## 1. Introduction

With the intensification of the livestock and poultry industry, large quantities of organic-rich animal manure urgently require effective resource utilization. Aerobic composting, owing to its operational simplicity, low cost, and the agronomic value of its end product, is widely recognized as one of the most effective strategies for the harmless treatment and resource recovery of livestock manure [1,2,3]. Under optimal ambient conditions, the composting process transitions through four discrete successive phases: mesophilic, thermophilic, cooling, and maturation. Mesophilic microorganisms first drive the heating, and thermophilic microorganisms then decompose refractory organic matter and inactivate pathogens at thermophilic temperature phases. After cooling, mesophilic microorganisms degrade lignocellulose and accumulate humus. A well-managed composting pile typically reaches the thermophilic phase (≥50 °C) within 1–3 days and completes the entire process in approximately 30–40 days, yielding a stabilized, pathogen-free end product. However, composting efficiency and product maturity are severely constrained in high-latitude regions and during winter due to low ambient temperatures. Under such conditions, microbial activity is suppressed, resulting in slow temperature rise and difficulty in maintaining the thermophilic stage. Consequently, organic matter degradation becomes incomplete, the composting cycle is prolonged, and the final product may fail to meet sanitation standards [4,5]. Previous studies have reported that under low-temperature conditions, the start-up stage of composting can extend beyond 25 days, while the duration of the thermophilic stage (>50 °C) may decrease by more than 50%, leading to insufficient pathogen inactivation and incomplete stabilization of organic matter [6,7]. Therefore, developing effective strategies to enhance composting performance under low-temperature conditions is of considerable practical importance.

Exogenous amendment strategies have been widely employed to enhance composting performance under low-temperature conditions. The introduction of specific microbial consortia can accelerate temperature rise, promote substrate decomposition, and regulate microbial community succession [5,8]. For instance, Tian et al. demonstrated that inoculation with psychrotolerant cellulose-degrading microorganisms during the co-composting of mushroom residues and sawdust significantly accelerated temperature elevation, enabling the compost pile to enter the thermophilic stage by day 3 with a peak temperature of 66.25 °C and a cellulose degradation rate of 40.85% [5]. Yin et al. reported that the addition of a composite microbial inoculant (PLC-8) to spent mushroom substrate composting advanced the onset of the thermophilic stage by 15 days [7]. In our previous studies, *Streptomyces griseorubens* JSD-1, isolated by our research group, exhibited strong lignocellulose-degrading capability [9]. Genome analysis revealed the presence of key lignocellulose-degrading genes, including laccase, exo-1,4-β-glucanase, and endo-1,4-β-glucanase [9]. This strain has also demonstrated the ability to enhance organic matter transformation and regulate bacterial community composition during rice straw–pig manure co-composting [10]. Additionally, *Arthrobacter nitroguajacolicus* LDT1-8 possesses strong cold tolerance and can maintain metabolic activity under low-temperature conditions.

In addition to microbial inoculation, the addition of readily degradable carbon sources represents another effective strategy for stimulating microbial activity during composting. Molasses, which is rich in soluble sugars such as sucrose and glucose, can provide an easily accessible carbon source for microorganisms, thereby facilitating rapid temperature elevation in composting systems [11,12]. Nevertheless, the combined effects of molasses and microbial inoculants on compost physicochemical properties, lignocellulose degradation, and microbial community succession remain insufficiently understood.

Therefore, this study conducted aerobic composting of cattle manure under winter low-temperature conditions to investigate the effects of molasses, the microbial inoculant JSD-1, the microbial inoculant LDT1-8, and their combined applications on the composting process. During the composting process, changes in temperature, physicochemical properties, lignin components and humus substances were monitored. At the same time, the succession pattern of microbial communities was analyzed using high-throughput sequencing technology. It is hypothesized that the combined addition of molasses and the two microbial inoculants would produce synergistic effects, significantly accelerating the temperature rise, prolonging the thermophilic stage, enhancing lignocellulose degradation and humification, and ultimately improving compost maturity under low-temperature conditions, compared with individual amendments or the control. In particular, we expected that molasses, as a readily degradable carbon source, would provide an initial energy boost to the cold-adapted inoculants (JSD-1 and LDT1-8), facilitating their metabolic activity, thereby leading to superior composting performance. This study clarified the mechanism of the composite amendment, providing a theoretical basis and technical support for improving the composting efficiency of livestock manure in cold regions.

## 2. Materials and Methods

### 2.1. Materials Preparation

The microbial strains used in this study were obtained from the culture collection of our research group, including Streptomyces griseorubens JSD-1 (CGMCC No. 5706) and *Paenarthrobacter nitroguajacolicus* LDT1-8 (CGMCC No. 31578).

Gause’s No.1 liquid medium consisted of KNO_3_ (1.0 g), MgSO_4_·7H_2_O (0.5 g), FeSO_4_·7H_2_O (0.01 g), K_2_HPO_4_ (0.5 g), NaCl (0.5 g), soluble starch (20.0 g) and distilled water (1 L). The pH value was adjusted to 7.2–7.4.

The CMC fermentation medium contained sodium carboxymethyl cellulose (CMC-Na, 10.0 g), peptone (5.0 g), yeast extract (1.0 g), and distilled water (1 L), with the pH value adjusted to 7.0–7.2.

### 2.2. Experimental Design

#### 2.2.1. Preparation of Solid Microbial Inoculants

The strain JSD-1 was first cultured in Gause’s No.1 liquid medium at 30 °C and 180 rpm for 3 days, which served as the liquid inoculum. The solid carrier was prepared by mixing peat and rice husk at a ratio of 1:1 (*w*/*w*) and supplementing with 0.4% corn starch, followed by sterilization via autoclaving. The sterile carrier was inoculated with JSD-1 liquid culture at 300–450 mL·kg^−1^ substrate. After thorough mixing, the mixture was incubated at 30 °C for 6 days under sealed conditions. When the substrate surface developed a silvery-gray appearance accompanied by an earthy odor, the material was dried and crushed to obtain the JSD-1 solid inoculant. The viable cell count of the inoculant was maintained at no less than 0.5 × 10^8^ CFU·g^−1^.

For LDT1-8, the liquid inoculum was prepared in CMC fermentation medium and incubated at 20 °C with shaking at 180 rpm for 2 days. The resulting culture was inoculated into the sterilized solid carrier at 300–450 mL·kg^−1^ and thoroughly mixed to produce the LDT1-8 solid inoculant (viable cell density ≥ 0.5 × 10^8^ CFU·g^−1^).

#### 2.2.2. Composting Experiment

Low-temperature aerobic composting experiments were conducted in January 2025 using 25 L composting reactors. Each reactor was fitted with an internal separation grid and filter cloth to retain solids and allow leachate collection, with a drainage valve installed at the base for leachate removal. Fresh cattle manure (moisture content 60.75%; C 52.82%; N 10.17%) was used as the composting substrate. Urea was added to adjust the initial C/N ratio to approximately 35:1, and the moisture content was adjusted to ~65%. Eight treatments were established: CK (no additive), T1 (molasses), T2 (JSD-1 inoculant), T3 (LDT1-8 inoculant), T4 (molasses + JSD-1 inoculant), T5 (molasses + LDT1-8 inoculant), T6 (JSD-1 inoculant + LDT1-8 inoculant), and T7 (molasses + JSD-1 inoculant + LDT1-8 inoculant). The total additive dosage was fixed at 2% (*w*/*w*, fresh weight basis) of the composting substrate. This dosage was selected based on preliminary laboratory-scale experiments [10,13], indicating that a 2% addition rate effectively promotes thermogenesis without causing excessive acidification or nutrient imbalance. Each treatment was performed in triplicate. The composting system was operated using passive aeration through natural ventilation combined with manual turning, without forced aeration. Compost piles were turned once per day during the mesophilic and thermophilic phases and once every two days during the cooling and maturation phases to maintain adequate oxygen supply and improve substrate homogeneity. Based on temperature dynamics, samples were collected on days 0, 1, 2, 4, 6, 9, and 12. Day 0 represented the initial substrate, days 1–4 corresponded to the thermophilic stage (≥50 °C), day 6 to the cooling stage (<50 °C), and days 9–12 to the maturation stage.

### 2.3. Analytical Methods

#### 2.3.1. Physicochemical Analyses

Compost temperature was monitored using a probe thermometer at 10:00 and 17:00 each day (Deli Group Co., Ltd., Ningbo, China). To account for spatial heterogeneity, measurements were taken at three locations (center, mid-point, and edge) within each pile. The mean value was recorded as the daily temperature. The standard deviation among the three measurement points was generally <1.5 °C, indicating relatively uniform heat distribution within the composting system. For pH values and electrical conductivity (EC) analysis, compost samples were extracted with deionized water at a solid–liquid ratio of 1:10 (*w*/*v*).

Total organic carbon (TOC) was determined using a combustion-based TOC/TN analyzer (multi N/C 3100, Analytik Jena AG, Jena, Germany), whereas total nitrogen (TN) was measured by the Kjeldahl method. The carbon-to-nitrogen ratio (C/N) was calculated as TOC/TN. Total phosphorus (TP) and total potassium (TK) were determined by inductively coupled plasma optical emission spectrometry (ICP–OES) following HNO_3_–H_2_O_2_ digestion. Organic matter (OM) was quantified using the loss-on-ignition method. The E4/E6 ratio was measured by UV–visible spectrophotometry [14]. The germination index (GI) was determined according to the standard method described in NY/T 525-2021 (Organic Fertilizer) [15].

#### 2.3.2. Lignocellulose and Humic Substance Fractions

Based on physicochemical performance, treatments T4, T5, T7, and the control (CK) were selected for further compositional analysis. Cellulose, hemicellulose, and lignin contents were determined using the Van Soest detergent fiber method, in which neutral detergent fiber (NDF), acid detergent fiber (ADF), and acid detergent lignin (ADL) were quantified to calculate the respective fractions [16]. Humic substance fractions were determined following the Chinese agricultural standard NY/T 1867-2010 (Determination of Soil Humus Composition) [17].

#### 2.3.3. Microbial Community Analysis

Compost samples from CK, T4, T5, and T7 collected on days 0, 1, 2, 4, 6, 9, and 12 were used for microbial community analysis. Total genomic DNA was extracted using the Omega Soil DNA Kit according to the manufacturer’s protocol (Omega Bio-tek, Inc., Norcross, GA, USA). DNA integrity was verified by 0.8% agarose gel electrophoresis, and DNA concentration was measured using a NanoDrop 2000 (Thermo Fisher Scientific, Wilmington, DE, USA).

For bacterial community analysis, the V3–V4 region of the 16S rRNA gene was amplified using primers 338F (5′-ACTCCTACGGGAGGCAGCA-3′) and 806R (5′-GGACTACHVGGGTWTCTAAT-3′). Fungal communities were amplified using the ITS5 (5′-GGAAGTAAAAGTCGTAACAAGG-3′) and ITS2 (5′-GCTGCGTTCTTCATCGATGC-3′) primer pair targeting the ITS region. PCR products were quantified using the Quant-iT PicoGreen dsDNA Assay Kit (Thermo Fisher Scientific, Wilmington, DE, USA) on a BioTek FLx800 Microplate Reader (BioTek Instruments, Inc., Winooski, VT, USA). Sequencing libraries were constructed using the Illumina TruSeq Nano DNA LT Library Prep Kit (Illumina, Inc., San Diego, CA, USA) and purified with AMPure XP beads (Beckman Coulter, Brea, CA, USA). Paired-end sequencing was performed on the Illumina MiSeq platform (Illumina, Inc., San Diego, CA, USA). Raw sequences were quality filtered and processed using Quantitative Insights Into Microbial Ecology (QIIME2) [18].

### 2.4. Data Analysis

Microbial diversity analyses were performed using QIIME2. Amplicon sequence variant (ASV) tables were used to calculate α-diversity indices, including Chao1, Observed species, Shannon, and Simpson indices. Sequencing depth adequacy was assessed using rarefaction curve analysis based on observed ASVs. As shown in Figure S1, rarefaction curves for all samples approached a clear plateau at approximately 15,000–20,000 sequences, indicating that the sequencing effort was sufficient to capture the majority of microbial diversity. No samples exhibited continuous upward trends at high sequencing depths, suggesting the absence of under-sequenced samples. Prior to downstream analyses, all samples were rarefied to an equal sequencing depth to minimize potential bias associated with uneven sampling effort. Community differences among samples were evaluated using principal coordinates analysis (PCoA).

Taxonomic composition at different classification levels was visualized using R (v4.2.0). Biomarker taxa were identified using linear discriminant analysis effect size (LEfSe) implemented in the Galaxy platform (https://galaxyproject.org, accessed 8 October 2025), with a Kruskal–Wallis test (*p* < 0.05) followed by linear discriminant analysis (LDA), and an LDA score threshold of 3.0. To control for false positives, *p*-values were adjusted using the Benjamini–Hochberg false discovery rate (FDR) correction. Pearson correlation coefficients among the top 50 ASVs (relative abundance > 0.01%) were calculated in R (v4.2.0). Co-occurrence networks were constructed based on robust correlations with an absolute correlation coefficient (|r|) > 0.6 and a significance level of *p* < 0.05. To reduce potential false positives, *p*-values were adjusted using the Benjamini–Hochberg false discovery rate (FDR) correction. Only statistically significant correlations were retained for network visualization using Gephi 1.01. Redundancy analysis (RDA) was conducted using the GenesCloud platform (https://www.genescloud.cn, accessed 8 October 2025) to evaluate relationships between microbial community structure and physicochemical parameters.

Other experimental data were processed and statistically analyzed using Microsoft Excel and GraphPad Prism (v8.0.2). Differences among treatments were evaluated by one-way ANOVA followed by Tukey’s multiple comparison test at a significance level of *p* < 0.05. Figures were generated using GraphPad Prism (v8.0.2).

## 3. Results

### 3.1. Changes in Physicochemical Properties During Composting

#### 3.1.1. Changes in the Temperature, pH, EC, E4/E6 and GI

**Figure 1:**
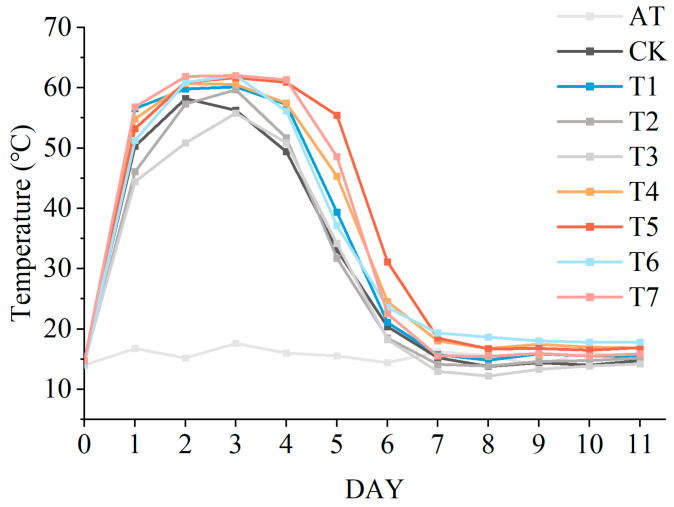
Changes in compost temperature under different treatments. CK, no additive; T1, molasses addition; T2, JSD-1 inoculant; T3, LDT1-8 inoculant; T4, molasses + JSD-1 inoculant; T5, molasses + LDT1-8 inoculant; T6, JSD-1 inoculant + LDT1-8 inoculant; T7, molasses + JSD-1 inoculant + LDT1-8 inoculant. AT: ambient temperature, the same below.

Composting temperature is a key indicator reflecting microbial metabolic activity and the progression of aerobic composting [19]. In this study, temperatures in all treatments increased rapidly during the initial stage (Figure 1). Although no significant differences in temperature were observed among treatments (*p* > 0.05), treatments amended with molasses (T1, T4, T5 and T7) reached maximum temperatures of 60.2, 60.6, 61.7 and 62.1 °C, respectively, all higher than that of CK (58.2 °C). This increase is likely associated with the combined effects of readily degradable carbon sources in molasses and microbial activity. These carbon sources, such as sucrose and glucose, rapidly supply substrates for microbial metabolism. Both inoculated and indigenous microorganisms contribute to substrate degradation and heat generation [12]. Moreover, the combined treatments of molasses and LDT1-8 inoculants maintained the thermophilic phase (≥50 °C) for 5 days, which was 2 days longer than that observed in CK. Similar effects have been reported in previous studies, where the addition of microbial inoculants accelerated composting initiation and temperature rise [7,20]. Importantly, the thermophilic duration in T4, T5 and T7 met the hygienic requirement for organic fertilizer production in China (GB 7959-2012). The prolonged thermophilic phase is beneficial for sanitation, facilitating the inactivation of pathogens and parasite eggs while promoting the initial degradation of lignocellulosic materials [21].

**Figure 2:**
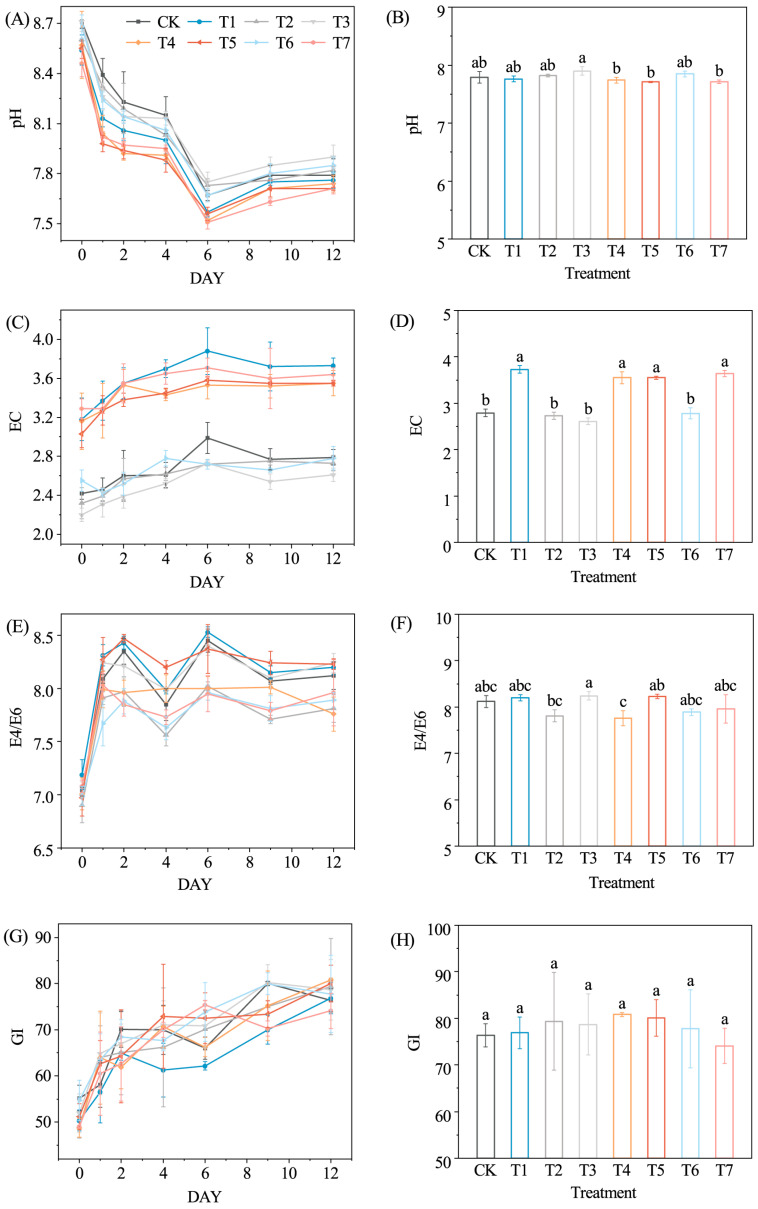
Changes in pH values (**A**), EC (**C**), E4/E6 (**E**), and GI (**G**) during composting under different treatments. pH values (**B**), EC (**D**), E4/E6 (**F**), and GI (**H**) of the final compost products under different treatments. Different lowercase letters indicate a significant difference among treatments (*p* < 0.05).

pH values are a key environmental factor regulating microbial community structure and metabolic activity during composting. In this study, pH values in all treatments decreased rapidly during the initial stage and reached a minimum on day 6, followed by a gradual increase to 7.7–8.0 (Figure 2A). This pattern is mainly associated with the accumulation of intermediate organic acids, such as acetic and pyruvic acids, produced during organic matter degradation [22,23]. Notably, the pH of T4 was lower than that of T2, and T5 was lower than T3 during the thermophilic phase (day 1~day 4), suggesting that the combined addition of molasses with JSD-1 or LDT1-8 enhanced microbial activity and accelerated acid-producing processes during organic matter degradation [9,10].

Electrical conductivity (EC) reflects the balance between microbial metabolism and ion release in the composting system [24]. EC values in all treatments increased during the early stage and peaked on day 6 before stabilizing (Figure 2C). T1, T4, T5, and T7 showed higher EC values than CK from the beginning of composting, which may be attributed to the input of soluble minerals such as potassium and calcium contained in molasses [25].

The E4/E6 ratio exhibited clear dynamic changes during composting. All treatments showed a marked increase in E4/E6 from day 0 to day 2 (Figure 2E), which may reflect microbial degradation of labile substrates and the accumulation of low-molecular-weight humic precursors [26]. Subsequently, E4/E6 values gradually decreased, indicating the progressive transformation of organic matter toward more complex humic substances. Treatments containing the JSD-1 inoculant (T2, T4, T6, and T7) showed lower E4/E6 values than CK on days 2 and 6 and remained lower by day 12. This trend, together with the observed increase in HA content as described in Section 3.2 (Lignocellulose Degradation and Humification), suggests a higher degree of humification. As an indirect indicator, the E4/E6 ratio reflects changes in molecular complexity rather than providing direct structural evidence. HA formation is largely associated with the condensation of phenolic compounds derived from lignin degradation with amino-containing precursors. In this study, the rapid increase in HA content in T4 and T7 during the thermophilic phase corresponded with the decrease in E4/E6, supporting the interpretation that the combined addition of molasses and JSD-1 may facilitate lignocellulose degradation and promote the transformation of FA into HA [27].

GI values increased continuously during composting, and the final products in all treatments exceeded 70% (Figure 2G), meeting the maturity requirement of the Chinese agricultural standard Organic Fertilizer (NY/T 525-2021). The GI values in the T4 and T7 treatments increased rapidly from the 6th to the 9th day, which was consistent with the sharp decrease in the E4/E6 ratio. The increase in GI values is not only related to the mineralization of easily degradable organic matter, but also to the complexation and fixation of toxic substances by humus [28].

#### 3.1.2. Changes in the Nutrient Content

**Figure 3:**
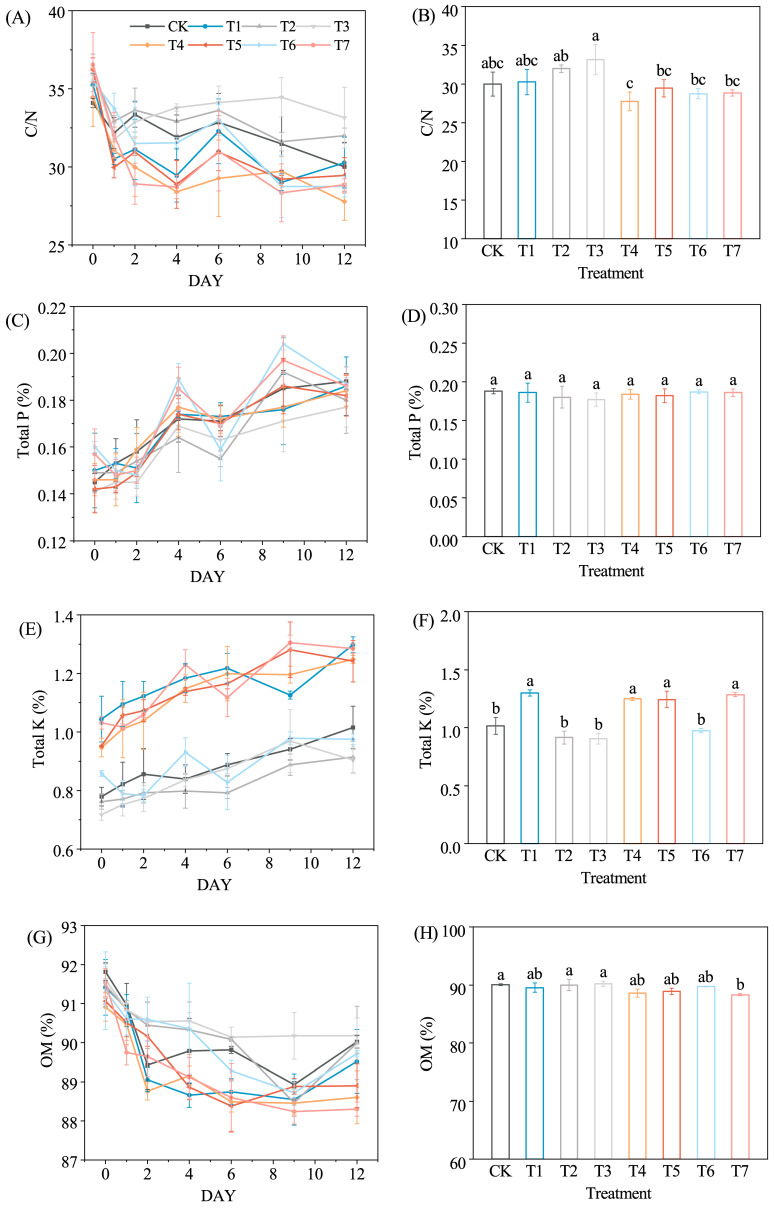
Changes in the C/N ratio (**A**), total phosphorus (TP) content (**C**), total potassium (TK) content (**E**), and organic matter (OM) content (**G**) during the composting process under different treatments. Final values of the C/N ratio (**B**), TP content (**D**), TK content (**F**), and OM content (**H**) in the compost products under different treatments. Different lowercase letters indicate a significant difference among treatments (*p* < 0.05).

The changes in C/N ratio and organic matter (OM) content are key indicators for evaluating organic matter degradation and compost maturity [29]. The C/N ratio in all treatments declined rapidly (Figure 3A). Compared with CK, T4, T5 and T7 exhibited larger reductions in C/N (19.41%, 18.64% and 21.08%, respectively) than CK (11.97%), indicating that the combined addition of molasses and microbial inoculants enhanced organic carbon mineralization and accelerated compost maturation. Similar effects have been reported in composting systems supplemented with polymicrobial inoculants at low temperatures [30]. A transient increase in the C/N ratio was observed in some treatments. Although nitrogen speciation was not directly measured, this pattern likely reflects a temporary imbalance in nitrogen transformation, wherein intensified ammonification and subsequent NH_3_ volatilization during the thermophilic phase outpace carbon mineralization [31]. Further investigation using detailed nitrogen speciation analyses is required to verify this mechanism. In addition, localized anaerobic microsites within the compost matrix may induce denitrification and N_2_O loss, further contributing to fluctuations in nitrogen dynamics [25].

TP and TK showed a gradual enrichment during composting (Figure 3E,G), mainly reflecting a concentration effect associated with organic matter mineralization, as the progressive loss of organic mass leads to the relative accumulation of stable mineral elements. The higher TK levels observed in molasses-amended treatments (T1, T4, T5, and T7) may partly be attributed to potassium supplied by molasses [32]. TP did not differ significantly among treatments, indicating that phosphorus changes were more closely related to the transformation of organic phosphorus within the compost matrix than to the direct input of external amendments [33]. A similar trend has been reported in apple wood and cattle manure composting, where TP variation was influenced by the balance between organic phosphorus mineralization and metal–phosphate precipitation [28].

Changes in OM further reflect the sequential utilization of substrates by microbial communities. Microorganisms preferentially degrade labile compounds such as sugars and proteins during the high-temperature phase before shifting to more recalcitrant substrates, including cellulose and hemicellulose [27]. Treatments amended with molasses (T1, T4, T5, and T7) showed greater OM reduction (Figure 3G), suggesting that the available carbon source stimulated early microbial activity. T7 achieved the highest OM degradation rate of 3.87%, which was significantly higher than that of CK (*p* < 0.05), indicating a synergistic effect between molasses and the microbial inoculants JSD-1 and LDT1-8 (Figure 3H). JSD-1 is capable of producing cellulolytic enzymes that enhance lignocellulose degradation [10], while LDT1-8 exhibits strong cold tolerance, facilitating rapid temperature rise and early decomposition during composting.

### 3.2. Lignocellulose Degradation and Humification

**Figure 4:**
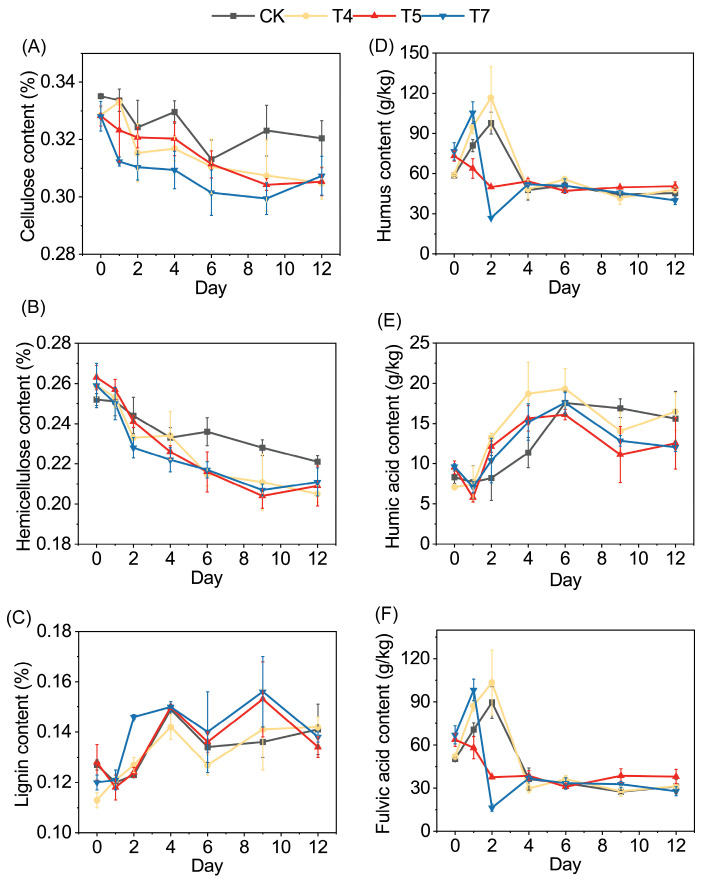
Dynamics of lignocellulose degradation and humification during the composting process under different treatments. (**A**) Cellulose, (**B**) hemicellulose, (**C**) lignin, (**D**) humic substances (HS), (**E**) humic acid (HA), and (**F**) fulvic acid (FA). No significant differences were detected among treatments (*p* > 0.05).

Different additive treatments regulated lignocellulose degradation and humification during composting. Cellulose and hemicellulose decreased gradually across treatments, whereas the relative lignin content showed a fluctuating increasing trend (Figure 4). Lignin was quantified using the Van Soest method and is expressed as a relative proportion; thus, its apparent increase reflects preferential polysaccharide degradation rather than a net accumulation. In the control (CK), the degradation rates of cellulose and hemicellulose were only 4.39% and 12.49%, respectively. The limited number of efficient lignocellulosic-degrading strains and the reduced activity of cryomicroorganisms may limit the secretion of extracellular degrading enzymes and the degradation of substrates, consistent with previous observations in low-temperature composting [34].

The addition of molasses and inoculants enhanced lignocellulose degradation. Relative to CK, cellulose degradation increased by 63.4%, 58.6%, and 44.0% in T4, T5, and T7, respectively, while hemicellulose degradation increased by 68.1%, 64.5%, and 49.3%. Faster early-stage degradation in T7 coincided with rapid microbial establishment, suggesting enhanced initial substrate transformation. Molasses likely stimulated microbial proliferation by supplying available carbon, whereas the introduced functional microbes may have increased the abundance of enzyme-producing populations, thereby enhancing extracellular enzymatic degradation of lignocellulose [35].

During the thermophilic phase, the relative proportion of lignin exhibited apparent enrichment, which can be attributed to the preferential degradation of polysaccharides and the inherent recalcitrance of aromatic structures to enzymatic attack [36,37]. A slight decline during the cooling and maturation phases likely resulted from the activity of mesophilic actinomycetes and fungi capable of producing oxidative enzymes such as laccases and peroxidases [38]. The observed lignin transformation during the late composting stage may be associated with the enrichment of Actinobacteria and auxiliary activity enzyme families (AA1 and AA3) [39].

Changes in humic substances reflected the dynamic balance between organic matter decomposition and humification during composting. The content of HS and FA increased rapidly at the initial stage, then declined and eventually stabilized at a relatively constant level. During the early stage of composting, microbial degradation of proteins and polysaccharides generated various humification precursors. Phenolic compounds, quinones, and other aromatic intermediates produced during these processes can undergo condensation reactions to form soluble humic fractions [12]. During the thermophilic phase, HS concentrations decreased, possibly because unstable HS fractions were mineralized, reutilized by microorganisms, or transformed into more polymerized fractions before structural stabilization occurred [40]. FA functions as an important transitional component during the humification process. The decline in FA occurred in T7, suggesting that the combined addition of molasses and inoculants may have promoted the conversion of humification intermediates into stable humic fractions, thereby accelerating the overall humification process.

HA gradually accumulated during composting. HA formation is associated with several microbially mediated reactions, including polyphenol oxidation and condensation, Maillard reactions, and the transformation of microbial residues [5]. In addition, active microbial metabolism during the thermophilic phase can promote the condensation and aromatization of organic intermediates, further facilitating the formation of stable humic structures [41]. Compared with CK, the HA content was higher in T4, T5 and T7 groups. The addition of exogenous inoculants may have accelerated the conversion of humus intermediates to stable humus components.

Pearson correlation analysis revealed clear associations among lignocellulose degradation, humification, and physicochemical parameters during composting (Figure S2). Cellulose and hemicellulose were strongly correlated, suggesting coordinated depolymerization of lignocellulose by microbial enzyme systems. Organic matter (OM) and C/N were positively correlated with lignocellulose composition, indicating that changes in these parameters are closely associated with lignocellulose dynamics during composting. In contrast, temperature was negatively correlated with OM and C/N. This pattern may reflect enhanced microbial activity and increased carbon mineralization at elevated temperatures, as reported in heat-regulated composting systems [42]. However, these correlations do not imply direct causality, and the underlying mechanisms require further investigation.

Clear relationships also emerged among humification indices. FA decreased as HA accumulated, a pattern consistent with the transformation of low-molecular-weight intermediates into more condensed humic structures [12]. Thermophilic functional microbes may further facilitate polymerization of organic intermediates into stable humic substances. Meanwhile, the GI values declined with increasing OM, C/N, and lignocellulosic components, suggesting that continuous degradation and humification gradually alleviated phytotoxicity and promoted compost maturation.

### 3.3. Microbial Diversity and Ecological Succession During Composting

#### 3.3.1. Biodiversity of Bacterial and Fungal Communities

The microbial community represents a central driver of organic matter transformation during aerobic composting. In this study, the diversity indices of bacterial communities (Shannon, Simpson and Chao1) exhibited a consistent pattern characterized by an initial decline followed by gradual recovery. The trends in *α*-and *β*-diversity were further supported by Supplementary Figure S3. This pattern reflects the environmental screening imposed by temperature shifts on microbial communities. During the initial heating phase, the community is dominated by fast-growing taxa that use available substrates and elevate metabolic activity, which raises composting temperature. As temperature rises, thermophilic conditions suppress part of the mesophilic microbiota, resulting in a temporary reduction in diversity. With the subsequent cooling phase, microorganisms capable of degrading more complex substrates gradually establish new ecological niches, leading to the recovery of community diversity. Such temperature-driven succession patterns are widely recognized in compost ecosystems [37]. Compared with the CK treatment, T4, T5 and T7 exhibited significantly lower bacterial diversity during the thermophilic phase (*p* < 0.0001). This decrease likely reflects intensified ecological competition following the addition of molasses and exogenous microbial inoculants, which rapidly enriched efficient degraders and reduced community evenness. With the degradation of available substrates, microbial diversity in the amended treatments recovered and eventually surpassed that of CK. More complex and functionally complementary microbial communities developed [41].

Fungal communities showed a more pronounced response to environmental fluctuations. Both Chao1 and Observed species indices declined markedly during the early stage of composting. The rapid temperature increase likely suppressed temperature-sensitive fungi, resulting in a temporary loss of diversity. As composting proceeded and conditions stabilized, thermotolerant fungal taxa gradually colonized the system. The Shannon and Simpson indices in the amended treatments were significantly lower than those in CK during the early stage (*p* < 0.0001). The rapid temperature rise promoted by molasses and microbial inoculation likely intensified environmental screening of fungal communities. PCoA analysis revealed clear separation between the amended treatments and CK from day 2 onward. Changes in substrate availability and microbial competition substantially reshaped fungal community structures [5].

#### 3.3.2. Taxonomic Composition of Bacterial and Fungal Communities

**Figure 5:**
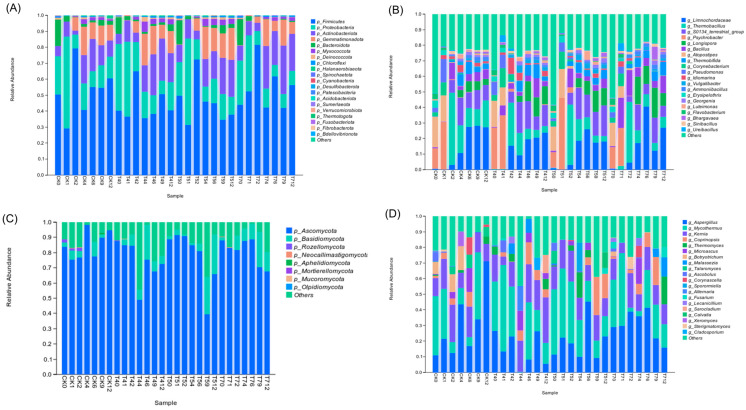
Taxonomic composition of microorganisms in different composts. (**A**) The phylum level composition of bacteria, (**B**) the genus level composition of bacteria, (**C**) the phylum level composition of fungi, and (**D**) the genus level composition of fungi.

At the phylum level, bacterial communities were predominantly composed of Firmicutes (49.09%), followed by Proteobacteria (19.71%), Actinobacteriota (15.16%), Gemmatimonadota (9.12%), and Bacteroidota (3.54%) (Figure 5A). Firmicutes remained dominant throughout the composting process, with their relative abundance increasing during the thermophilic phase and declining during maturation. This pattern is consistent with the well-known capacity of many Firmicutes members to produce cellulases and hemicellulases, thereby facilitating the degradation of plant residues under high-temperature conditions [7]. In particular, the T7 treatment rapidly enriched Firmicutes to 81.58% by day 2, suggesting a potential association between Firmicutes dominance and accelerated lignocellulose transformation.

Proteobacteria, typically associated with the utilization of labile substrates, exhibited a transient increase during the early stage, followed by a decline under thermophilic conditions and a subsequent recovery during the cooling phase [3]. Actinobacteriota showed progressive enrichment during the later stages, especially in T4, T5, and T7, where their relative abundance was significantly higher than in CK (*p* < 0.0001). Notably, the abundance reached 30.35% in T7 on day 9, approximately 2.5 times that observed in CK (12.40%). This trend is in agreement with previous observations that Actinobacteriota are frequently associated with the transformation of more recalcitrant organic matter during compost maturation [28].

At the genus level, *Limnochordaceae* showed enrichment in the T7 treatment during the thermophilic stage (Figure 5B), consistent with previous reports linking this lineage to lignocellulose degradation [26]. *Bacillus* maintained consistently higher relative abundance in the T4, T5, and T7 treatments compared with CK, with a peak of 19.54% in T7 on day 1. Previous studies have shown that *Bacillus* species play a key role in accelerating compost degradation processes [38]. In addition, *Longispora* exhibited sustained enrichment during the cooling and maturation stages. This genus has previously been associated with higher seed germination index (GI) values and reduced phytotoxicity in compost systems [43].

Fungal communities were dominated by Ascomycota across all treatments with an average relative abundance of 79.57% (Figure 5C). Previous studies have shown that members of this phylum produce a wide range of cellulolytic and hemicellulolytic enzymes [27]. Basidiomycota were significantly enriched in T4, T5 and T7 across multiple stages [28]. These results indicate potential shifts in fungal community structure associated with substrate transformation. At the genus level (Figure 5D), *Aspergillus* and *Mycothermus* were among the dominant taxa, particularly in the thermophilic and cooling phases, which were likely associated with efficient cellulose and hemicellulose degradation [26]. In addition, genera such as *Kernia* became more abundant during the later stages in T4 and T5 [44]. These compositional changes suggest that the combined amendments reshaped fungal community succession patterns during composting.

As this study is based on amplicon sequencing, these interpretations represent potential associations rather than direct functional evidence. Future studies using metagenomic or transcriptomic approaches are needed for validation.

#### 3.3.3. LEfSe Analysis and Microbial Correlation Network

**Figure 6:**
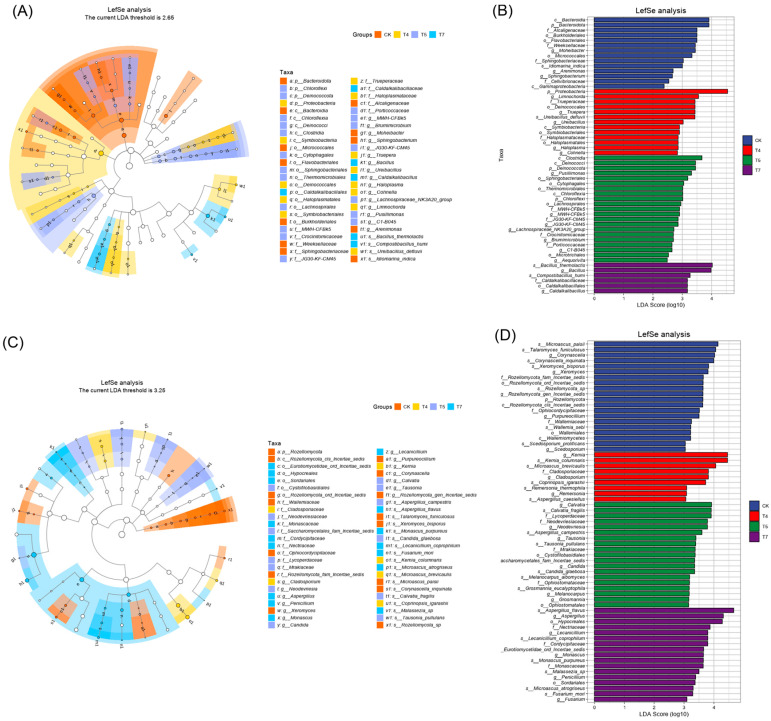
LEfSe analysis of microbial communities in composting. (**A**) Cladogram of differential bacterial taxa. (**B**) LDA scores of bacterial biomarkers. (**C**) Cladogram of differential fungal taxa. (**D**) LDA scores of fungal biomarkers.

Only taxa with LDA scores > 3.0 and FDR-adjusted *p*-values < 0.05 were considered as significant biomarkers. LEfSe analysis identified 226 bacterial and 159 fungal differential taxa, indicating that the addition of molasses and microbial inoculants significantly altered microbial community composition (Figure 6). In the CK treatment, key bacterial biomarkers included Bacteroidota-related taxa and Proteobacteria-associated lineages, which are typically involved in polysaccharide degradation and nitrogen cycling. In T4, taxa affiliated with Limnochorda and the radiation-resistant phylum Deinococcota (including *Trueperaceae*, *Deinococcales* and *Truepera*) were significantly enriched. These organisms exhibit strong stress tolerance and may promote organic matter mineralization and humic substance formation. In T5, *Pusillimonas* and *Sphingobacteriales* were enriched, both of which are associated with complex organic matter transformation. The T7 treatment was characterized by the enrichment of Bacillus-related taxa such as *Compostibacillus* and *Caldalkalibacillaceae*, which possess strong cellulolytic capabilities.

Co-occurrence network structures are presented in Figure S4, further revealing that the addition of molasses and microbial inoculants substantially reshaped microbial interaction patterns during composting. Compared with CK, the amended treatments exhibited higher network density and average degree. This increase in network complexity suggests that the combined amendments promoted closer metabolic cooperation and substrate exchange within the microbial community, thereby facilitating more efficient organic matter transformation [45]. In addition, the modularity of microbial networks increased in the amended treatments. Higher modularity generally reflects the formation of relatively independent ecological niches, which may enhance community stability and functional specialization during composting [2].

At the taxonomic level, the central nodes of the networks differed markedly among treatments. In the CK network, Limnochordaceae and Atopostipes acted as major hubs, indicating that indigenous lignocellulose-degrading bacteria dominated microbial interactions under natural composting conditions. In contrast, *Flavobacterium* emerged as a key node in the T4 network, a genus known for its ability to degrade complex organic substrates and promote humification processes. In the T5 network, *Ammoniibacillus* and *Pseudomonas* occupied central positions, suggesting their important roles in extracellular enzyme secretion and protein degradation, which may provide precursors for humic substance formation. Notably, *Thermobifida* became a core hub in the T7 network, highlighting the importance of thermophilic cellulolytic microorganisms in driving lignocellulose degradation during the thermophilic phase [46]. Fungal networks exhibited similar restructuring patterns. In CK, *Microascus* and *Coprinopsis* were identified as key nodes associated with lignocellulose degradation. In the T5 treatment, *Candida* became a central taxon. In T7, network hubs shifted to thermophilic fungi such as *Aspergillus* and *Thermomyces*, key decomposers of plant residues under high temperatures.

### 3.4. Correlation Between Microbial Communities and Environmental Parameters

Redundancy analysis (RDA) revealed clear associations between microbial community composition and environmental variables during composting (Figure S5). Permutation tests confirmed that the RDA models were statistically significant (*p* < 0.05). For bacterial communities, the first two axes (RDA1 and RDA2) explained 49.77–70.59% and 15.15–29.52% of the total variation across treatments, respectively, with cumulative contributions ranging from 78.97% to 88.92%. For fungal communities, RDA1 and RDA2 explained 48.34–62.83% and 13.6–27.4% of the total variation, respectively, with cumulative contributions ranging from 72.82% to 82.4%. Humic fractions (HA, FA, and HS) together with lignocellulosic components (HEM and LIG) formed the major environmental gradients influencing microbial succession.

In bacterial communities, thermophilic taxa such as Bacillus were positively correlated with temperature and humic fractions. These taxa have a clear ecological advantage during the thermophilic phase and contribute to organic matter degradation and humic precursor formation [22]. In addition, *Limnochordaceae*, *Longispora*, and S0134_terrestrial_group showed positive correlations with GI, TP, TK, and lignin, indicating potential involvement in lignocellulose degradation and nutrient release. For fungal communities, *Mycothermus* was positively associated with cellulose, hemicellulose, and organic matter, reflecting its role in structural carbohydrate decomposition. In contrast, *Aspergillus* showed strong correlations with lignin, humic acid, and germination index, suggesting its contribution to lignin transformation and humification processes [47]. In the combined amendment treatments, relationships between humic components and fungi such as *Aspergillus*, *Microascus*, and *Kernia* became more pronounced. The amendments favored the enrichment of functional lignocellulose-degrading fungi.

Building upon the enhanced composting performance described above, the economic feasibility of the combined treatment (molasses + JSD-1 + LDT1-8) should be considered for large-scale application. Molasses is an inexpensive and widely available by-product of the sugar industry, and its low application rate limits additional costs. Similarly, microbial inoculants can be produced at relatively low cost through large-scale fermentation. The improved composting performance observed in this study may contribute to reduced processing time and improved product quality, thereby partially offsetting the input costs. However, a detailed cost–benefit analysis was not conducted in this study, and further evaluation under large-scale conditions is required to fully assess the economic viability of this approach in cold regions.

## 4. Conclusions

This study used cattle manure as the primary substrate to investigate the effects of molasses, *Streptomyces griseorubens* JSD-1, *Arthrobacter nitroguajacolicus* LDT1-8, and their combinations on aerobic composting under low-temperature conditions. The results showed that the combined addition of molasses and microbial inoculants significantly improved the heating performance and compost maturity. In particular, the combined treatment (T7) exhibited a faster temperature increase, a longer thermophilic phase, and more efficient organic matter degradation, accompanied by improved nutrient quality. In terms of lignocellulose degradation, the composite initiator enhanced the decomposition of cellulose and hemicellulose. The T7 treatment also promoted the transformation of FA into HA, thereby optimizing the humification process. Microbial community analysis revealed that although the combined addition slightly reduced microbial diversity during the thermophilic stage, it selectively enriched key functional taxa. Among bacteria, Actinobacteriota, *Bacillus*, *Longispora*, and *Limnochordaceae* were significantly enriched, while in the fungal community, Basidiomycota as well as thermophilic genera such as *Mycothermus* and *Kernia* showed increased relative abundance. Redundancy analysis indicated that FA, HS, HA, and HEM were the primary factors driving bacterial community succession, whereas lignin, total phosphorus, C/N ratio, and humic components were the key environmental variables shaping fungal community structure. This study demonstrates that the synergistic coupling of readily available carbon sources and functional microbial inoculants can reshape microbial ecological succession and accelerate lignocellulose degradation and humification.

These findings provide a practical microbial regulation strategy for improving composting efficiency in cold regions. Future research should focus on optimizing additive dosages and evaluating this strategy under field-scale conditions in cold regions. Integrating multi-omics approaches (e.g., metagenomics or metatranscriptomics) would further elucidate the functional mechanisms underlying microbial succession and lignocellulose transformation. In addition, economic feasibility and the long-term stability of the resulting compost products warrant further investigation to support practical application.

## Data Availability

The original contributions presented in this study are included in the article/Supplementary Material. Further inquiries can be directed to the corresponding author.

## Supplementary Materials

The following supporting information can be downloaded at: https://www.mdpi.com/article/10.3390/microorganisms14051077/s1, Figure S1: Rarefaction curves of microbial communities across all samples based on observed ASVs. The curves approached saturation at approximately 15,000–20,000 sequencing reads, indicating sufficient sequencing depth to capture the majority of microbial diversity; Figure S2: Correlation analysis of different indicators in different sample dimensions (A). Correlation analysis of different indicators at different time dimensions (B); Figure S3: Microbial diversity in different compost treatments. (A) Bacterial α-diversity and (B) fungal α-diversity. Principal coordinate analysis (PCoA) of β-diversity for (C) bacterial communities and (D) fungal communities; Figure S4: Co-occurrence networks of dominant microbial genera during composting. (A–D) Bacterial networks in the CK, T4, T5, and T7 treatments, respectively. (E–H) Fungal networks in the CK, T4, T5, and T7 treatments, respectively. Network construction was based on correlations satisfying |r| > 0.6 and *p* < 0.05, ensuring that only robust associations were included in the analysis; Figure S5: Redundancy analysis (RDA) of microbial communities and environmental factors during composting. (A–D) RDA of bacterial communities in the CK, T4, T5, and T7 treatments, respectively. (E–H) RDA of fungal communities in the CK, T4, T5, and T7 treatments, respectively. Environmental factors include: TE (temperature), pH, EC (conductivity), TP (total phosphorus), TK (total potassium), C/N ratio, OM (organic matter), GI (germination index), E4/E6, HS (soluble humus), HA (humic acid), FA (fulvic acid), HEM (hemicellulose), CEL (cellulose), LIG (lignin).
