# Supplementary material for: Carbon-Stimulated Bioaugmentation Enhances Thermogenesis, Lignocellulose Degradation, and Humification in Low-Temperature Cattle Manure Composting
Source: Microorganisms. 2026 May 10;14(5):1077. doi: 10.3390/microorganisms14051077 (PMC13209998; doi:10.3390/microorganisms14051077)
Supplement: Supplementary file 1 [file microorganisms-14-01077-s001.zip › microorganisms-4265004-supplementary.pdf]

Supplementary data

Carbon-stimulated bioaugmentation enhances thermogenesis, lignocellulose degradation, and humification in low-temperature cattle manure composting

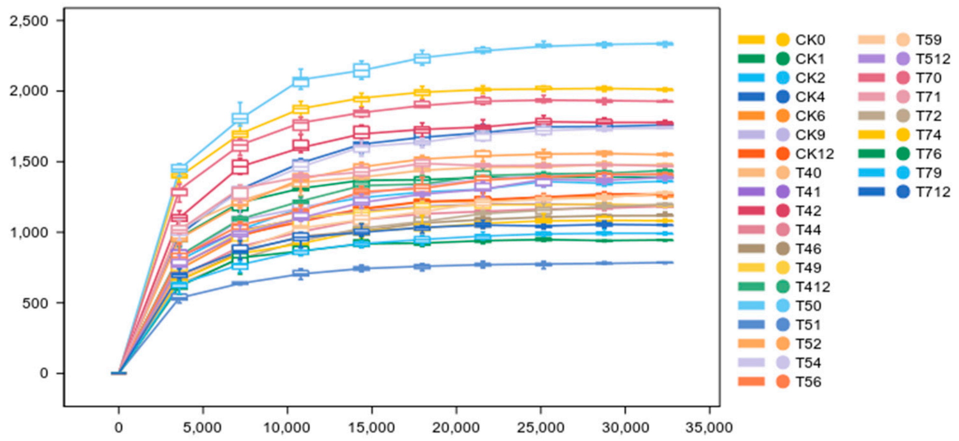

Figure S1. Rarefaction curves of microbial communities across all samples based on observed ASVs. The curves approached saturation at approximately 15000–20000 sequencing reads, indicating sufficient sequencing depth to capture the majority of microbial diversity.

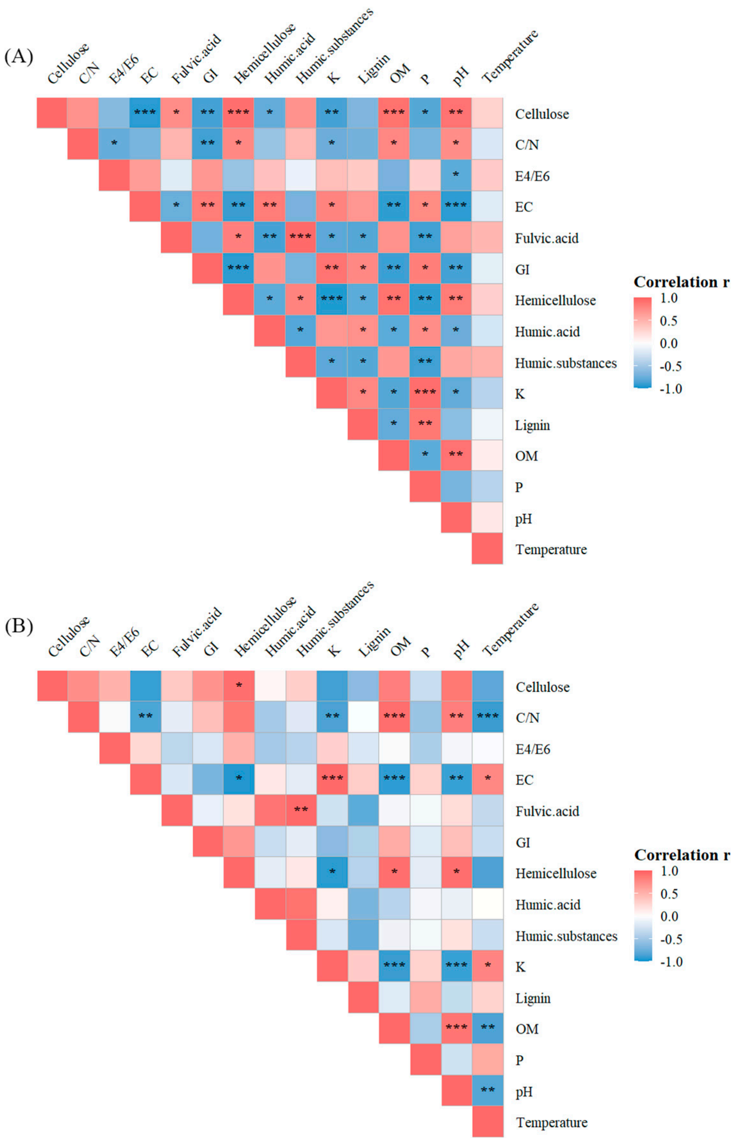

Figure S2. Correlation analysis of different indicators in different sample dimensions (A). Correlation analysis of different indicators at different time dimensions (B).

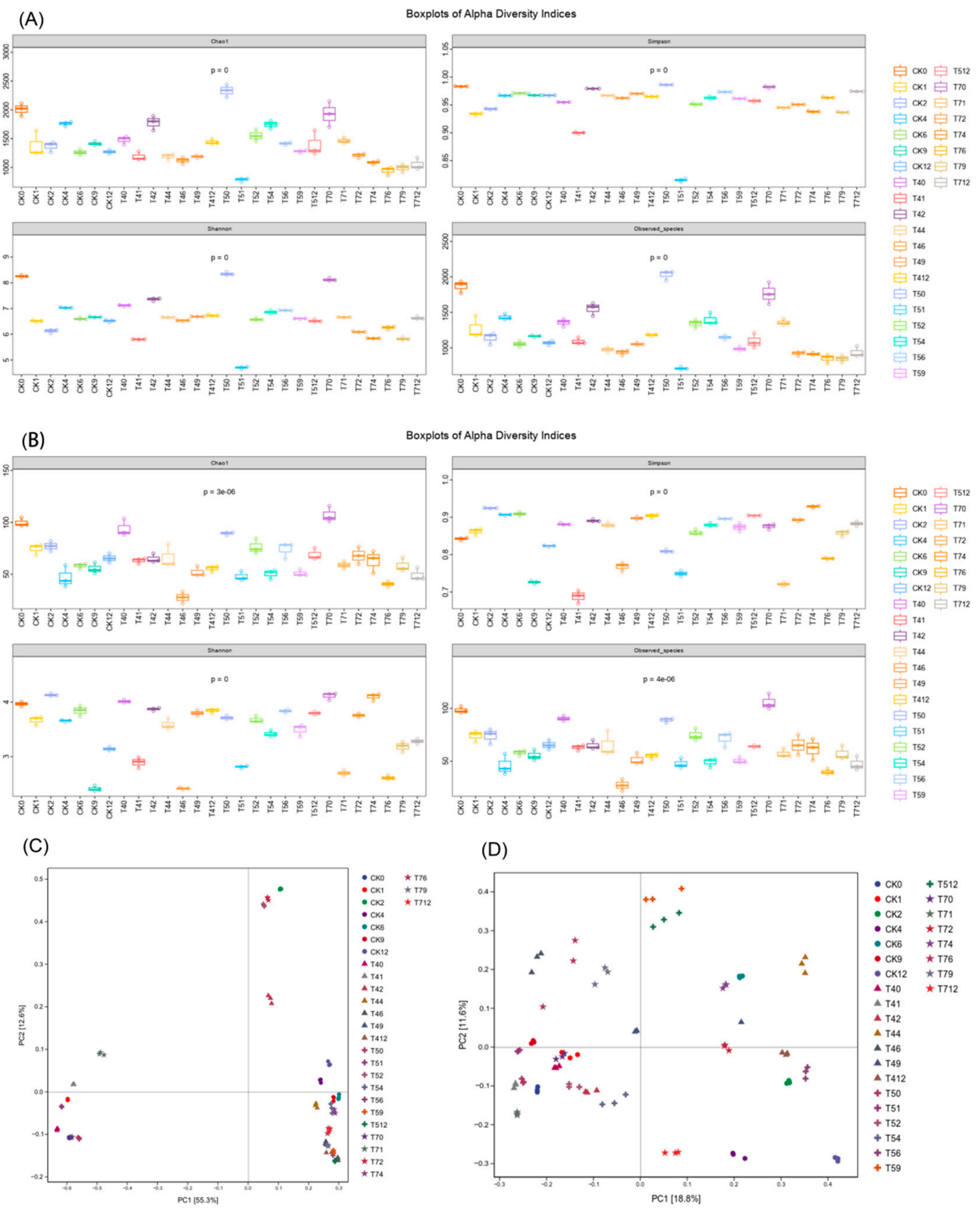

Figure S3. Microbial diversity in different compost treatments. (A) Bacterial α-diversity and (B) fungal α-diversity. Principal coordinate analysis (PCoA) of β-diversity for (C) bacterial communities and (D) fungal communities.

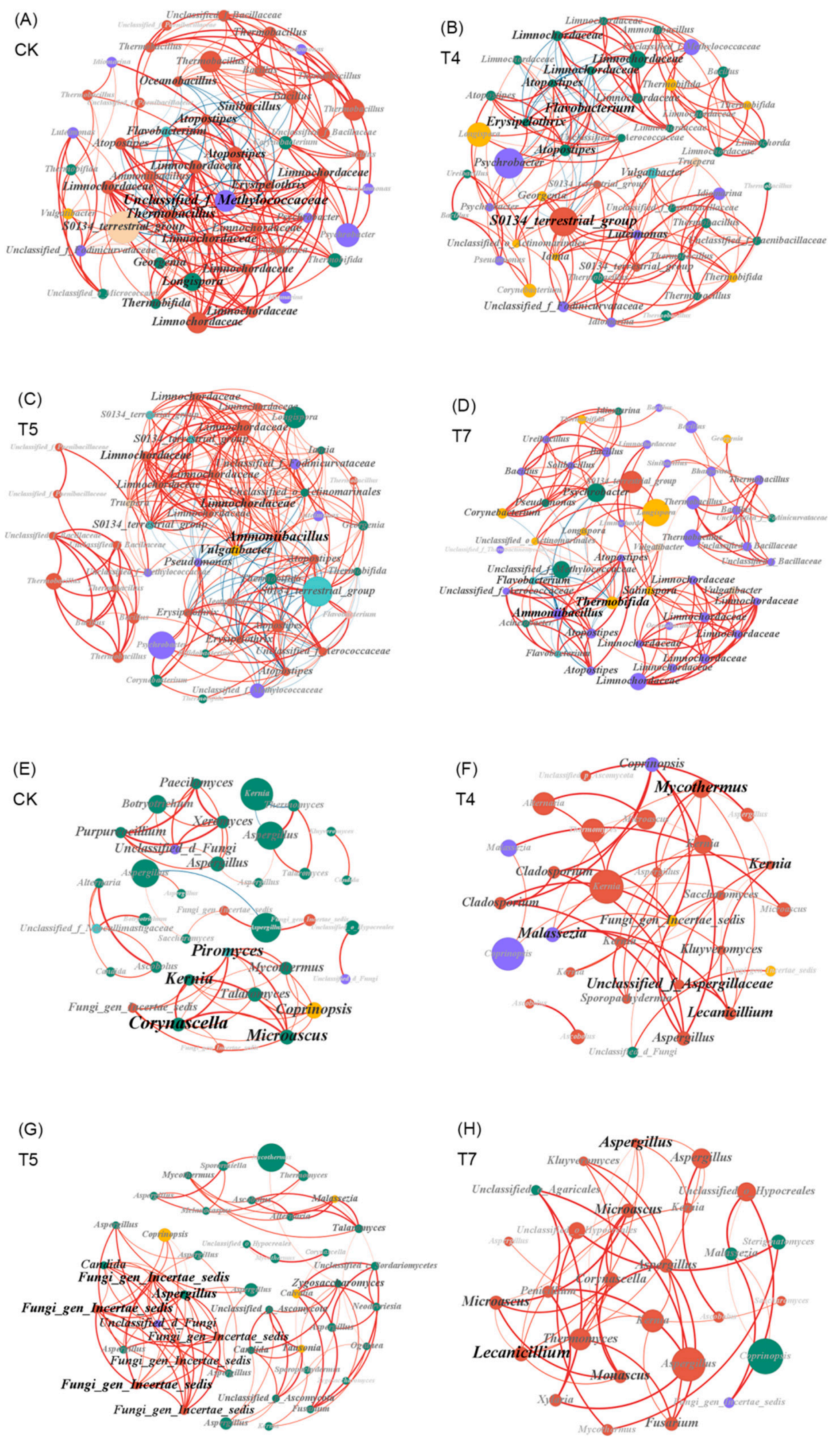

Figure S4. Co-occurrence networks of dominant microbial genera during composting. (A–D) Bacterial networks in the CK, T4, T5, and T7 treatments, respectively. (E–H) Fungal networks in the CK, T4, T5, and T7 treatments, respectively. Network

construction was based on correlations satisfying $|r| > 0.6$ and $p < 0.05$, ensuring that only robust associations were included in the analysis.

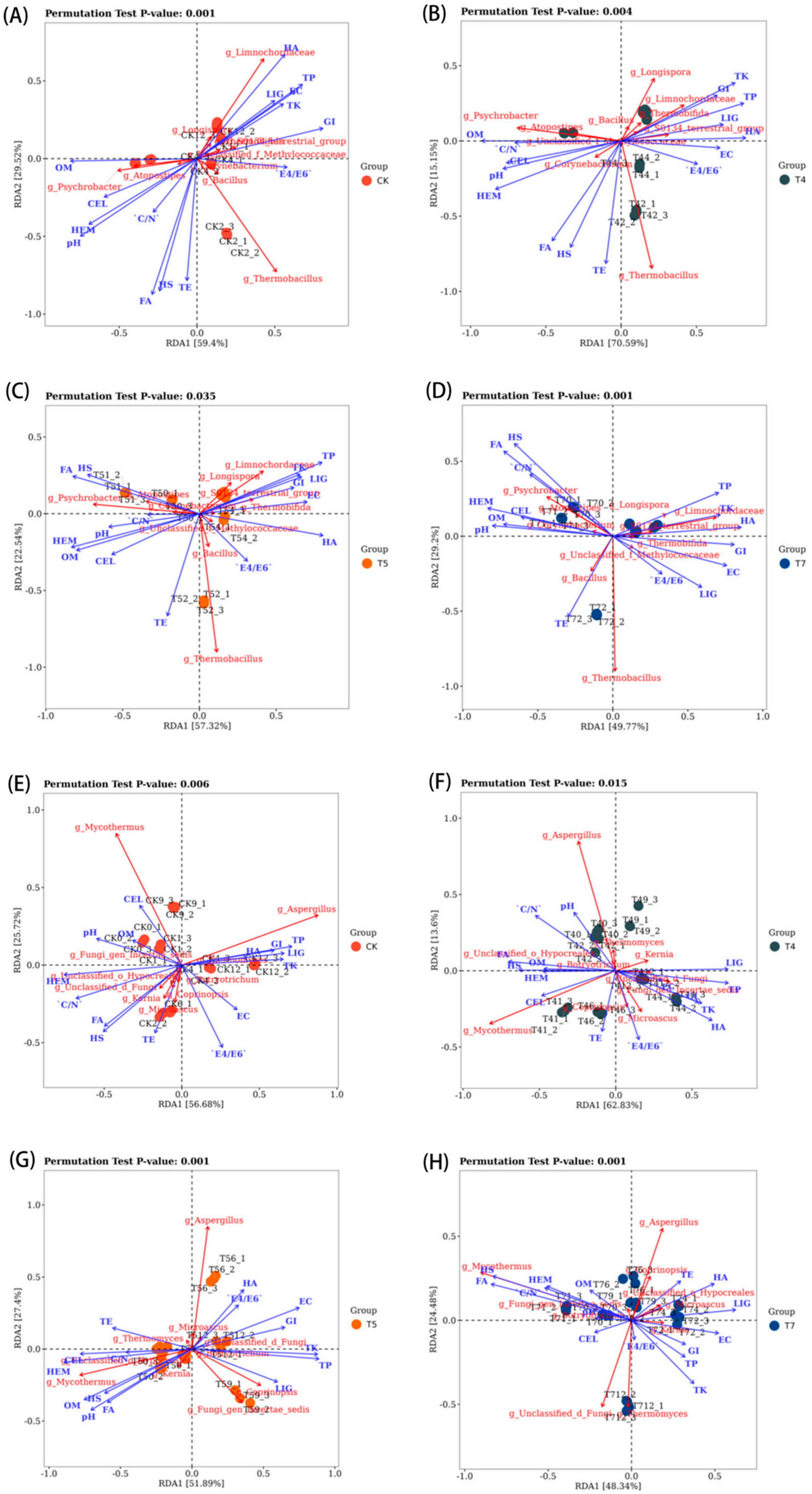

(A)
Permutation Test P-value: 0.001
RDA1 [59.4%]
RDA2 [29.52%]
Group
CK
(B)
Permutation Test P-value: 0.004
RDA1 [70.59%]
RDA2 [15.15%]
Group
T4
(C)
Permutation Test P-value: 0.035
RDA1 [57.32%]
RDA2 [22.54%]
Group
T5
(D)
Permutation Test P-value: 0.001
RDA1 [49.77%]
RDA2 [29.2%]
Group
T7
(E)
Permutation Test P-value: 0.006
RDA1 [56.68%]
RDA2 [25.72%]
Group
CK
(F)
Permutation Test P-value: 0.015
RDA1 [62.83%]
RDA2 [13.6%]
Group
T4
(G)
Permutation Test P-value: 0.001
RDA1 [51.89%]
RDA2 [27.4%]
Group
T5
(H)
Permutation Test P-value: 0.001
RDA1 [48.34%]
RDA2 [24.48%]
Group
T7

Figure S5. Redundancy analysis (RDA) of microbial communities and environmental factors during composting. (A–D) RDA of bacterial communities in the CK, T4, T5, and T7 treatments, respectively. (E–H) RDA of fungal communities in the CK, T4, T5, and T7 treatments, respectively. Environmental factors include: TE (temperature), pH, EC (conductivity), TP (total phosphorus), TK (total potassium), C/N ratio, OM (organic matter), GI (germination index), E4/E6, HS (soluble humus), HA (humic acid), FA (fulvic acid), HEM (hemicellulose), CEL (cellulose), LIG (lignin).
